# A Population Pharmacokinetic Modelling Approach to Unravel the Complex Pharmacokinetics of Vincristine in Children

**DOI:** 10.1007/s11095-022-03364-1

**Published:** 2022-08-19

**Authors:** A. Laura Nijstad, Wan-Yu Chu, Evelien de Vos-Kerkhof, Catherine F. Enters-Weijnen, Mirjam E. van de Velde, Gertjan J. L. Kaspers, Shelby Barnett, Gareth J. Veal, Arief Lalmohamed, C. Michel Zwaan, Alwin D. R. Huitema

**Affiliations:** 1 Department of Clinical Pharmacy, University Medical Center Utrecht, Heidelberglaan 100, 3584 CX Utrecht, the Netherlands; 2 Department of Pharmacology, Princess Máxima Center for Pediatric Oncology, Heidelberglaan 25, 3584 CS Utrecht, the Netherlands; 3 Department of Clinical Pharmacy, University Medical Center Utrecht, Internal postal D.00.204, Postbus 85500, 3508 GA Utrecht, The Netherlands; 4 Department of Pharmacy & Pharmacology, Netherlands Cancer Institute, Plesmanlaan 121, 1066 CX Amsterdam, the Netherlands; 5 Princess Máxima Center for Pediatric Oncology, Heidelberglaan 25, 3584 CS Utrecht, The Netherlands; 6 Julius Center for Health Sciences and Primary Care, University Medical Center Utrecht, Utrecht, The Netherlands; 7 Emma Children’s Hospital, Pediatric Oncology, Amsterdam UMC, Vrije Universiteit Amsterdam, 1105 AZ Amsterdam, The Netherlands; 8 Newcastle University Centre for Cancer, Newcastle University, Newcastle upon Tyne, NE2 4HH UK; 9 Utrecht Institute for Pharmaceutical Sciences, Utrecht University, Universiteitsweg 99, 3584 CG Utrecht, the Netherlands; 10 Department of Pediatric Oncology, Erasmus MC-Sophia Children’s Hospital, Dr. Molewaterplein 40, 3015 GD Rotterdam, the Netherlands

**Keywords:** oncology, pediatric, pharmacokinetics, population pharmacokinetics

## Abstract

**Background:**

Vincristine, a chemotherapeutic agent that extensively binds to β-tubulin, is commonly dosed at 1.4–2.0 mg/m^2^ capped at 2 mg. For infants, doses vary from 0.025–0.05 mg/kg or 50–80% of the mg/m^2^ dose. However, evidence for lower doses in infants compared to older children is lacking. This study was conducted to unravel the complex pharmacokinetics of vincristine, including the effects of age, to assist optimal dosing in this population.

**Methods:**

206 patients (0.04–33.9 years; 25 patients < 1 years), receiving vincristine, with 1297 plasma concentrations were included. Semi-mechanistic population pharmacokinetic analyses were performed using non-linear mixed effects modelling.

**Results:**

A three-compartment model, with one saturable compartment resembling saturable binding to β-tubulin and thus, saturable distribution, best described vincristine pharmacokinetics. Body weight and age were covariates significantly influencing the maximal binding capacity to β-tubulin, which increased with increasing body weight and decreased with increasing age. Vincristine clearance (CL) was estimated as 30.6 L/h (95% confidence interval (CI) 27.6–33.0), intercompartmental CL (Q) as 63.2 L/h (95%CI 57.2–70.1), volume of distribution of the central compartment as 5.39 L (95%CI 4.23–6.46) and of the peripheral compartment as 400 L (95%CI 357–463) (all parameters correspond to a patient of 70 kg). The maximal binding capacity was 0.525 mg (95%CI 0.479–0.602) (for an 18 year old patient of 70 kg), with a high association rate constant, fixed at 1300 /h and a dissociation constant of 11.5 /h.

**Interpretation:**

A decrease of vincristine β-tubulin binding capacity with increasing age suggests that young children tolerate higher doses of vincristine.

**Supplementary Information:**

The online version contains supplementary material available at 10.1007/s11095-022-03364-1.

## Introduction

Vincristine is used in the chemotherapeutic treatment of various pediatric malignancies. Its effect is caused by binding to tubulin and inhibiting microtubule formation, causing arrest of the cell at metaphase. Treatment with vincristine is mainly hampered by risk of developing vincristine induced peripheral neuropathy (VIPN). VIPN pathogenesis involves nerve cell mitochondria, endothelium and microtubules and has been shown to be dose-dependent [1, 2]. Younger children have been suggested to exhibit a lower risk of developing VIPN compared to adolescents, despite a higher dose per kg body weight administered, which underlines potential differences in pharmacokinetics (PK) and pharmacodynamics in the younger patient population [3].

Vincristine is usually dosed based on body surface area (BSA) (doses vary from 1.4–2.0 mg/m^2^) [4]. Because of the dose-dependent VIPN, the absolute dose is capped to a maximum of 2 mg [4]. However, clear evidence for this maximum dose in children is lacking. Additionally, commonly used dose reductions in infants are not evidence based. For infants, several dosing regimens are used in current practice. Doses vary from 0.025–0.05 mg/kg or 50–80% of the usual dose per BSA [5–11], but none of these reductions are based on literature. Theoretically, younger children could be at risk for lower vincristine clearance values, due to incomplete maturation of cytochrome p450 (CYP) 3A4, however, findings on age-related differences in PK of vincristine in infants and children are not conclusive.

Recently, Barnett *et al.* did not report significant differences in BSA-normalized vincristine clearance values between infants and older children, apart from a trend towards lower clearance in neonates (0–4 weeks) as compared to infants (1–12 months) [12]. They showed that doses of < 0.05 mg/kg resulted in significantly lower area under the curve (AUC) values than observed in infants and children receiving doses of ≥ 0.05 mg/kg or 1.5 mg/m^2^, showing that dose reductions to for example 0.025 mg/kg in infants could lead to underexposure. In a recently published in-depth literature review, based on these findings and the results of other PK studies that did not find a relationship between age and PK [13–21], we concluded that infants should be administered doses of 0.05 mg/kg or 1.5 mg/m^2^ [11].

Even though age-related differences in the PK of vincristine have not been found in published studies, Lee *et al.* proposed that there is a fivefold higher β-tubulin binding capacity in children compared to adults [22]. Using a physiologically based pharmacokinetic (PBPK) modeling approach, they suggested that binding to β-tubulin in healthy tissue could play a key role in vincristine distribution, which might explain differences in toxicity. An increased fraction of the vincristine dose bound to β-tubulin in healthy tissue may lead to lower amounts of free vincristine and thus a lower risk of VIPN. Indeed, it is well known that vincristine binds to β-tubulin. Moreover, β-tubulin is abundant in thrombocytes, and, decades ago, both *in vitro* and *in vivo* studies showed that vincristine rapidly binds to thrombocytes, so it is hypothesized that thrombocyte levels could also have an effect on vincristine distribution [23–29].

This current study was conducted to unravel the complex PK of vincristine, including the effects of age, using a semi-mechanistic population PK modelling approach. Unravelling the complex PK of vincristine in (very) young children, alongside key clinical pharmacology data recently published in this area [12], will promote more rational vincristine dosing in this patient population.

## Methods

### Patients and Sampling

A prospective observational study was performed in Princess Máxima Center for Pediatric Oncology in the Netherlands. Patients up to the age of 18 years with a central venous line *in situ* were eligible for inclusion after written informed consent was obtained. No restrictions for types of tumors or malignancies were formulated, but patients with Down syndrome were excluded. Ethical approval by the institutional Medical Ethics Committee of the Erasmus MC was obtained (NL63037.078.18). The data generated from this study were combined with data from an ongoing prospective observational study in 20 clinical cancer centers across the UK. In the UK study, patients with Ewing sarcoma up to 24 years of age with a central venous line *in situ* were eligible for inclusion after written informed consent was obtained. Patients with a glomerular filtration rate < 60 mL/min/1.73m^2^ were excluded. Ethical approval by the National Research Ethics Service committee North East-Newcastle and North Tyneside 1 was obtained (EudraCT 2013–000,052-17). Beside these two prospectively collected cohorts, data from three historical cohorts previously described by Lee *et al*. [22] (n = 24; only UK patients were included), van de Velde *et al.* [30] (n = 37) and Barnett *et al*. [12] (n = 26) were included in this analysis. All previous studies included patients up to the age of 18 years.

All patients were treated with vincristine as standard of care, with doses according to local protocols. Doses, varying from 1–2 mg/m^2^ with a maximum of 2 mg, with specific reductions for infants, were administered either as bolus or 1 h infusion.

In total, 4–8 blood samples per patient were collected at various time points. Vincristine plasma concentrations were quantified using a previously described high-performance liquid chromatography tandem mass spectrometry (LC–MS/MS) method [31] or a validated LC–MS assay developed in Newcastle [32], with lower limits of quantification (LLOQ) of 0.25 ng/mL and 0.50 ng/mL respectively. Vincristine plasma concentrations of the Princess Máxima Center for Pediatric Oncology study were quantified using a validated LC–MS/MS method using 200 µL human plasma, with a LLOQ of 0.10 ng/mL [33]. First samples below LLOQ were included using ½ of the LLOQ value.

When data on covariates (age, body weight (BW) and height) were missing, values were imputed based on UK growth charts [34] and known variables. For all cases where age was missing, BW and height were documented, therefore, these values were used to find the corresponding age in the growth charts (using median BW and height curves). In the cases where BW and height were missing, age and BSA were available. The age and BSA were used to find the corresponding height, and this value and the BSA were used to calculate the BW, using the Du Bois Eq. [35].

### Model Development

For the structural model, two- and three-compartment models with first order elimination were tested.

Saturable binding to β-tubulin was implemented by incorporating the maximal binding capacity (Bmax) in the differential equation as follows:

1 $$\frac{dA(bound)}{dt}={k}_{on}\times A\left(Vc\right)\times \left(1-\frac{A\left(bound\right)}{{B}_{max}}\right)-{k}_{off}\times A(bound)$$

where k_on_ is the association rate constant, k_off_ is the dissociation rate constant, A(Vc) is the amount of vincristine in the central compartment Vc, A(bound) is the amount of vincristine bound to β-tubulin and Bmax is the maximal binding capacity to β-tubulin. Bmax was estimated. See Supplementary Table S1 for differential equations of other compartments.

Interindividual variability (IIV) was evaluated for all PK parameters, and implemented as follows:

2 $${P}_{i}= {P}_{pop}\times {e}^{({\eta }_{i})}$$

where *P*_*i*_ is the individual parameter estimate for individual *i*, *P*_*pop*_ is the typical population parameters estimate, and *η*_*i*_ is assumed to be normally distributed with a mean of zero and a variance of *ω*^*2*^.

Since data of multiple cycles of therapy were available, interoccasion variability (IOV) was implemented similarly as IIV, with each dose and subsequent sampling defined as a separate occasion. This variability was evaluated for clearance parameters and Bmax to diagnose potential time-dependent trends and to allow for random unaccounted variability between dosing moments.

Residual unexplained variability was evaluated as a proportional error model or as a combination of a proportional and additive error model.

### Covariate Analysis

The influence of patient-specific factors for variability in PK parameters were evaluated following structural model development. Allometric scaling was applied to implement the impact of body weight on PK parameters with a fixed exponent of 0.75 resp. 1 for clearances resp. volumes of distribution. PK parameters were normalized to a BW of 70 kg [36]. Other assessed covariates included age and thrombocyte levels, using a power function, normalizing to an age of 18 years and a thrombocyte level of 300 × 10^9^/L, respectively.

### Model Evaluation

Discrimination between models was guided by physiological plausibility, goodness-of-fit (GOF) plots, precision of parameter estimates and change in objective function value (dOFV). A drop of ≥ 3.84 points, corresponding to a P < 0.05 (χ^2^-distribution with 1 degree of freedom (df)), was considered a significant improvement. The adequacy of the models was assessed by GOF plots and visual predictive checks (VPC) [37]. The sampling importance resampling (SIR) procedure was used for the assessment of parameter precision [38].

### Software

Nonlinear mixed-effects modeling was performed using NONMEM (version 7.3.0, ICON development Solutions, Ellicott City, MD, USA) and Pearl-speaks-NONMEM (PsN, version 4.9.0) with First-Order Conditional Estimation with interaction (FOCE-I) as estimation method [39, 40]. Pirana (version 2.9.9) was used as graphical user interface for NONMEM [41]. R (version 3.4.3) was used for data handling and visualization [42].

## Results

### Patients and Sampling

In total, 206 patients with a median age of 8.3 years (range 0.04–33.9) were included. Detailed patient characteristics are presented in Table I. 25 patients, with 25 vincristine cycles and 88 samples, were younger than 1 years of age (7 patients 0–3 months; 8 patients 3–6 months; 4 patients 6–9 months; 6 patients 9–12 months). In total, 1297 samples were available, of which 30 samples were below the LLOQ. Supplementary Figure S1 displays the observed plasma concentrations over time.

**Table I:** Patient Characteristics (Median (range), Unless Specified Otherwise)

	N = 206
Available data	
Total no. of occasions	253
Total no. of PK samples [n]	1297
No. of occasions per patient	1 (1–5)
No. of samples per occasion	5 (1–8)
Patient characteristics	
Age, years	8.3 (0.04–33.9)
No. of patients 0–1 yrs [n]	25
Actual body weight, kg	27.1 (2.9–126.0)
Female sex [n (%)]	98 (48%)
Thrombocyte levels	
Available occasions [n (%)]	137 (54%)
Thrombocyte levels, × 10^9^/L	224 (5–1063)
Not available occasions [n (%)]	116 (46%)
Vincristine treatment	
Dose, mg	1.6 (0.1–2.0)
Dose, mg/m^2^	1.4 (0.4–2.5)
Dose, mg/kg	0.05 (0.02–0.09)
Infusion duration [n]	
Bolus	214
15–113 min	39

*PK* Pharmacokinetic(s)

In total, for 8 patients the age was missing and for 2 patients BW and height were missing and were imputed based on UK growth charts. All these patients came from UK studies.

### Model Development

The base model that best described the data was found to be a three-compartment model with first order elimination. Allometric scaling using BW was *a priori* included on all PK parameters. Following structural model development, a saturable compartment was incorporated, resembling saturable binding to β-tubulin and thus, saturable distribution, to test the hypothesis of Lee *et al.* [22]. They hypothesized that binding to β-tubulin has a significant impact on the PK of vincristine. This third, saturable compartment was incorporated as a compartment, driven by the concentration in the central compartment. Adjustment of the base three-compartment model to a three-compartment model containing one saturable compartment, resulted in a drop in OFV of 80 points. This model was parameterized in terms of volume of distribution of the central (Vc) and peripheral (Vp) compartment, clearance from the central compartment (CL) as well as intercompartmental CL between Vc and Vp (Q), Bmax, the association rate constant (k_on_) and dissociation rate constant (k_off_). k_on_ was considered to be too fast to estimate adequately, so was fixed at 1300 /h (the value that resulted in the lowest OFV). The model was further optimized by adding IIV on CL, Q, Vc, Vp, k_on_ and k_off_ and IOV on Bmax. No trends in IOV on Bmax *vs*. dosing occasions or age were observed.

### Covariate Analysis

Subsequently, various covariates were tested for their influence on PK parameters and Bmax. In the PBPK model of Lee *et al.* [22], age was found to be a significant covariate for β-tubulin expression, defined as Bmax in our model. Furthermore, we expect Bmax to be dependent on BW, based on allometric scaling principles. For this reason, BW and age were tested as covariates on Bmax. Firstly, BW was included in Bmax using a power function with an estimated exponent. The exponent was estimated to be 0.707. This was thought to be a result of a combined, opposite effect of BW and age, where an increasing BW would lead to an increase in Bmax (allometric principles), but where an increase in age would lead to a decrease in Bmax (hypothesis Lee *et al.* [22]). For this reason, the exponent on BW was fixed to 1 (in accordance with allometric scaling for volumes of distribution) and age was included as covariate using a power function (normalization to a patient of 18 years), with an estimated exponent. This resulted in an exponent of -0.199 for age.

Furthermore, thrombocyte levels were tested as covariate on Bmax. Several studies showed that vincristine binds to thrombocytes, which is hypothesized to be related to tubulin, since β-tubulin isoforms are abundant in human thrombocytes [29]. Data on thrombocyte levels were not available for 46% of the occasions. When thrombocyte levels were not available, a thrombocyte count of 300 × 10^9^/L was imputed, plus IIV to allow for variability on this imputed value. An IIV of around 30% was found. However, adding thrombocyte levels as covariate on Bmax resulted in unstable models with divergent OFV values, very sensitive to initial estimate changes. In addition, the IOV on Bmax did not decrease and IIV’s on other parameters increased. Therefore, thrombocyte levels were not included as covariate on Bmax in the final model.

**Fig. 1:**
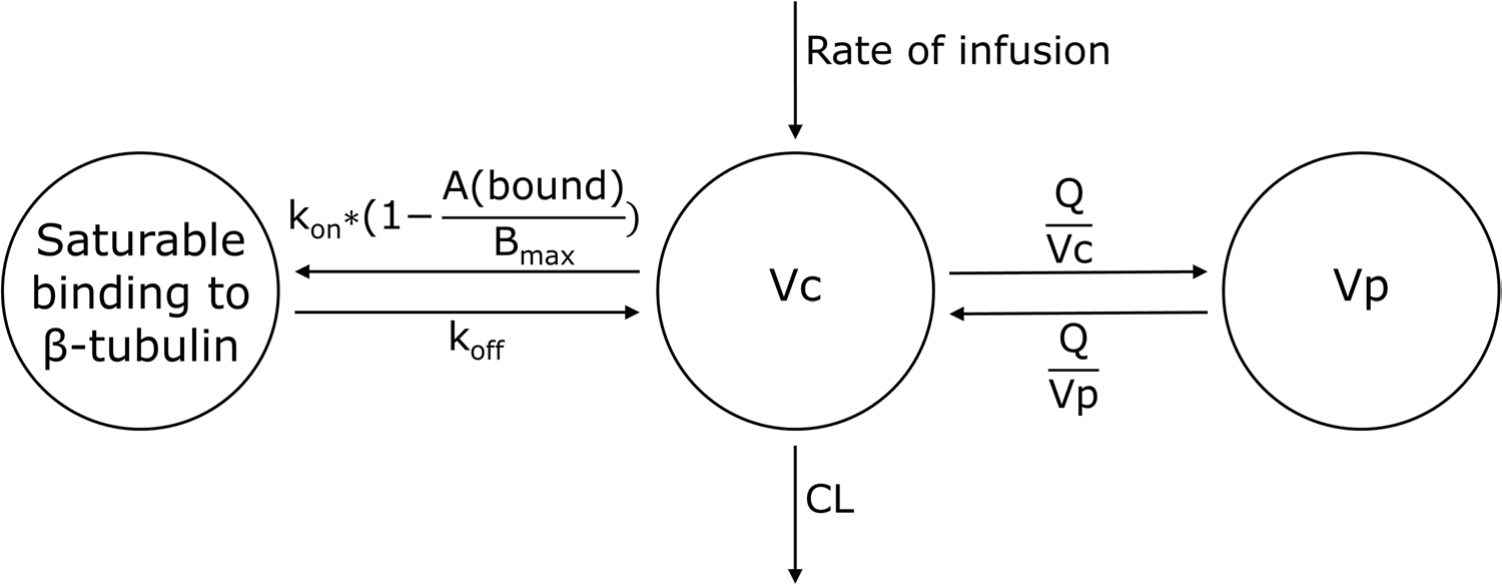
Graphical representation of the final model for vincristine. k_on_ is driven by the amount of vincristine, bound to tubulin (A(bound)) and Bmax. Bmax Maximal binding capacity; CL Clearance; k_off_ Dissociation rate constant; k_on_ Association rate constant; Q Intercompartmental clearance; Vc Vincristine central compartment; Vp Vincristine peripheral compartment.

**Fig. 2:**
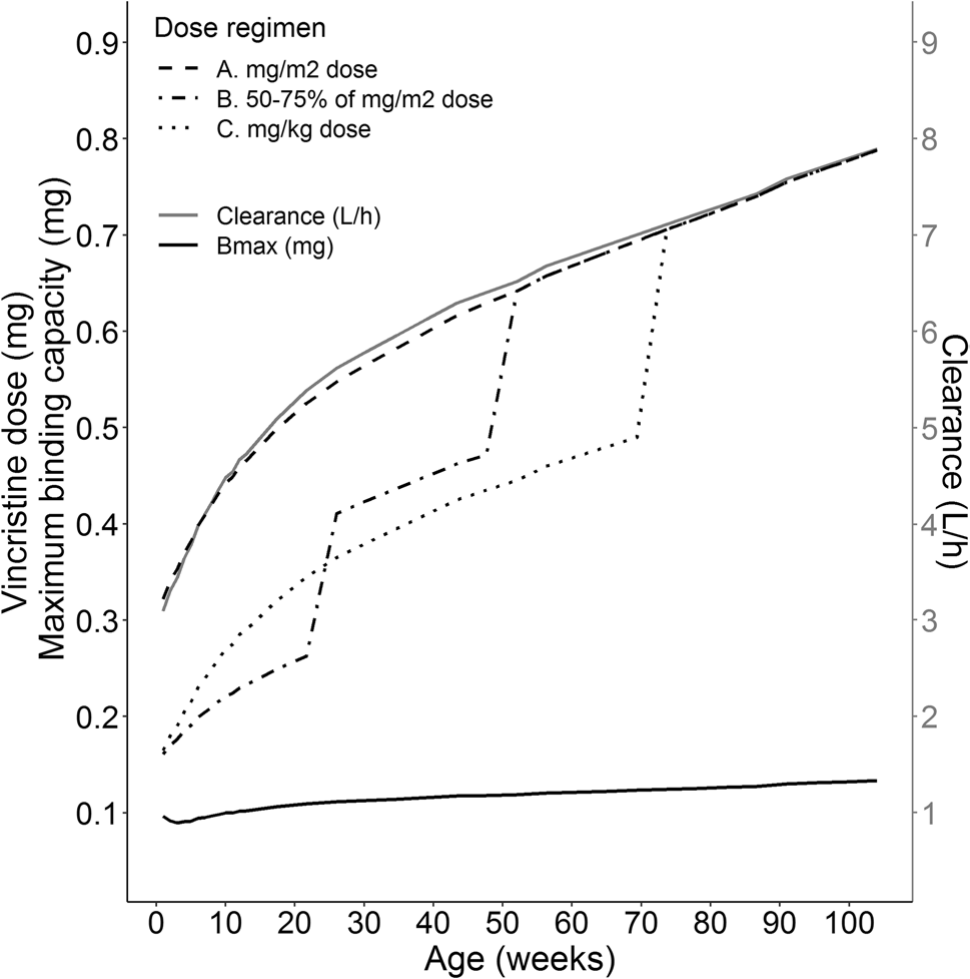
Vincristine clearance (solid grey line), maximum binding capacity (solid black line) and absolute vincristine dose for three different infant dosing regimen over age: A. All ages: 1.5 mg/m^2^ (dashed line). B. Children < 6 months: 50% of BSA dose (0.75 mg/m^2^); Children 6–11 months: 75% of BSA dose (1.125 mg/m^2^); Children ≥ 12 months: 1.5 mg/m^2^ (dashdotted line). C. Children < 10 kg: 0.05 mg/kg/day; Children ≥ 10 kg: 1.5 mg/m^2^ (dotted line).

A graphical representation of the final model is presented in Fig. 1. Final PK parameters estimates are displayed in Table II. Figure 2 displays the typical Bmax and CL vs. age for patients until the age of 2 years. Data was based on typical weight and height values according to WHO growth charts [34]. Absolute doses according to three different dosing regimens were included:

A. All ages: 1.5 mg/m^2^
B. Children < 6 months: 50% of BSA dose (0.75 mg/m^2^); Children 6–11 months: 75% of BSA dose (1.125 mg/m^2^); Children ≥ 12 months: 1.5 mg/m^2^
C. Children < 10 kg: 0.05 mg/kg/day; Children ≥ 10 kg: 1.5 mg/m^2^

**Table II:** Vincristine PK Parameters Estimates of the Final Model

Parameter	Estimate	95% CI
CL_70kg_ (L/h)	30.6	27.6 – 33.0
Q_70kg_ (L/h)	63.2	57.2 – 70.1
Vc_70kg_ (L)	5.39	4.23 – 6.46
Vp_70kg_ (L)	400	357 – 463
Bmax_18yrs,70 kg_ (mg)	0.525	0.479 – 0.602
k_on_ (/h)	1300 fixed	
k_off_ (/h)	11.5	9.2 – 14.5
Age on Bmax	-0.199	-0.304 – -0.090
IIV CL (%)	47.7	41.0 – 54.3
IIV Q (%)	38.1	26.2 – 49.0
IIV Vc (%)	122.5	98.7 – 158.3
IIV Vp (%)	57.1	48.8 – 69.7
IIV k_on_ (%)	126.5	108.7 – 147.8
IIV k_off_ (%)	24.1	11.1 – 33.8
IOV Bmax (%)	59.1	50.7 – 66.1
Proportional residual error (%)	30.1	28.9 – 31.4

*Bmax* Maximal binding capacity; *CI* Confidence interval obtained by sampling importance resampling; *CL* Clearance; *IIV* Interindividual variability; *IOV* Interoccasion variability; *k*_*off*_ Dissociation rate constant; *k*_*on*_ Association rate constant; *PK* Pharmacokinetic(s); *Q* Intercompartmental clearance; *Vc* central compartment; *Vp* peripheral compartment

Bmax corresponds to a subject of 18 years weighing 70 kg, other population estimates correspond to a subject weighing 70 kg and are adjusted to an individual value using allometric scaling

### Model Evaluation

The model performance was checked through GOF plots. Looking at population and individual predictions, conditional weighted residuals vs. plasma concentration and time after dose, no trends, or signs for over- of underprediction have been found (Supplementary Figure S2). Furthermore, the VPC showed no signs for structural over- of underprediction (Supplementary Figure S3). The PK parameters of the patients with imputed age or BW and height were not markedly different.

## Discussion

This study successfully implemented saturable binding to β-tubulin in a population PK model of vincristine in children. A three-compartment model, including one saturable compartment, was found to best describe the available data from 206 patients. This saturable compartment resembles saturable binding to β-tubulin and thus, saturable distribution of vincristine.

Two covariates were identified to account for variability in the binding capacity to β-tubulin. BW was included as covariate to Bmax using allometric principles. Additionally, age was added as covariate using a power function. BW and age were found to have an opposite effect on Bmax. By using allometric scaling for volumes of distribution, a rise in BW resulted in an increase in Bmax, while we found that a higher age led to a decrease in Bmax. This is in line with the findings of Lee *et al.* [22]. With their PBPK analyses they showed that there is a fivefold higher β-tubulin binding capacity in children compared to adults.

Clinical information on the difference in β-tubulin exposition between children and adults is not available, but it has been shown that immunohistochemical distribution of β2-tubulin was higher in tissue of neonates compared to older children and adults, and that the expression decreased with increasing age [43]. The same is to be expected for other β-tubulin isotypes, since tubulin in microtubules play an important role in cell division, which is more prevalent in children. A higher β-tubulin expression in younger children is most likely the reason for a higher β-tubulin binding capacity of vincristine. However, evidence for the differences in β-tubulin expression between neonates and older children remains missing. Future studies should address β-tubulin expression of various β-tubulin isotypes in different types of tissue and different age categories.

A higher β-tubulin binding capacity leads to a faster drop of the vincristine plasma concentration and lower amounts of free vincristine present in the central compartment thus a lower risk of VIPN, since we assume that free vincristine is able to distribute to peripheral tissue, where it causes VIPN. In children, with a higher β-tubulin binding capacity, a lower amount of free vincristine is available to distribute to peripheral tissue, while in adults, with a lower β-tubulin binding capacity, the amount of free vincristine is higher as well as the risk of developing VIPN. This hypothesis is consistent with the findings that younger children seem to tolerate higher doses with regards to development of VIPN compared to older children and adults [1, 3].

These findings also raise the question whether children should be administered higher doses than adults to achieve the same effect. Children are usually treated with doses of 1.5–2.0 mg/m^2^, with a maximum of 2 mg. Essentially, the capped dose comes into play for patients with a BSA > 1.3 m^2^. It is unclear to what extend and over what age range the risk of developing VIPN is lower than in adults, so definitive advice for changing the maximum dose for children cannot be given. For younger children, however, we could make some remarks based on the current PK study and previous research. As mentioned previously, infants are treated with doses varying from 0.025–0.05 mg/kg or 50–80% of the usual dose per BSA [5–11]. Barnett *et al.* [12] showed that doses of < 0.05 mg/kg result in significantly lower AUC values than observed in infants and children receiving doses of ≥ 0.05 mg/kg or 1.5 mg/m^2^. These exposure data, combined with our current results, strongly suggest that dose adjustments for infants may not be justified.

A major concern with current dosing approaches for vincristine, is that using different dosing regimens for infants leads to disproportional increases in the dose when the patient reaches a specific age or weight (see Fig. 2). While this widely used rudimentary approach to dosing is a concern for all drugs, it is of particular concern for a drug such as vincristine, for which no age-related differences in CL have been found. In the current study, we show that younger children have a higher β-tubulin binding capacity for vincristine. However, absolute Bmax values (taking BW and age into account) in patients up to 2 years of age, displayed in Fig. 2, do not seem to change markedly over time, except for the first weeks of age. Furthermore, Fig. 2 visually shows that a dose regimen of 1.5 mg/m^2^ follows the curve of vincristine CL with increasing age. From a pharmacokinetic perspective, we would suggest administering the full mg/m^2^ (e.g. 1.5 mg/m^2^) dose to infants, except for neonates of 0–4 weeks (0.05 mg/kg according to Barnett *et al.* [12]).

Increasing the dose should, however, be done with caution. Besides VIPN, other adverse reactions, like vocal cord paralysis, respiratory distress or constipation, frequently occur. Preferably, a clinical trial in young patients investigating the exposure and toxicity profiles under the proposed mg/m^2^ dosing regimen compared to the mg/kg dose is performed, before changing the dose in infants.

It would be interesting to perform simulations to explore different vincristine dosing regimens in patients of different ages in order to find the best dosing strategy for every patient. However, it is not known which PK parameter represents vincristine effectivity and toxicity. A parameter related to vincristine in the central compartment (like AUC or C_max_) might not be the right way to compare doses between different groups, since vincristine does not show its effect in the central compartment. It would be interesting if future studies would address this important topic.

Furthermore, in order to decrease the IOV on Bmax, we aimed to look into the effect of thrombocyte levels on the β-tubulin binding capacity. We did not find an effect of thrombocytes levels on Bmax or the IOV of Bmax. This is probably due to missing thrombocyte counts in a large part of the dataset. Moreover, since β-tubulin is present in all cells (all types of blood cells as well as cells in peripheral tissue) [25, 27, 44, 45], it is possible that thrombocytes account for just a small part of the β-tubulin expression throughout the body. This will be studied further using a PBPK modelling approach. In addition, it is possible that the β-tubulin binding capacity is dependent on tumour type or disease state. Tumour cells also express β-tubulin, so tumour type and/or disease state could influence vincristine distribution [23, 43]. The saturable compartment should therefore be interpreted as vincristine binding to all β-tubulin that is available. The current dataset did not contain disease type or status, so this was not tested as covariate yet.

A limitation of the current study is that the data did not include the exact amount of β-tubulin in patients, since we were not able to measure β-tubulin exposition. We have explained the saturable distribution as being vincristine binding to β-tubulin, however, alternative explanations could be made. We have looked into other developmental changes that could explain the age-related findings of the current study, but did not find other pharmacological rationales.

Another limitation relates to the fact that the effect of CYP3A4/5-inductors and –inhibitors on the PK of vincristine was not studied, since information on the use of CYP3A4/5-inductors and –inhibitors was not available. Also, genetic variations in CYP3A4/5, which can vary with race, were not taken into account in the current study. Moreover, the metabolic capacity of CYP3A4/5 change during the first years of life. However, it is to be expected that variations in activity of metabolising enzymes CYP3A4/5 only effect vincristine CL, and does not influence vincristine distribution, which is the main topic of the current study. Furthermore, previous research did not find an effect of CYP3A4/5 polymorphisms on vincristine PK [16].

## Conclusion

Vincristine binding to β-tubulin was found to be dependent of body weight and age. β-tubulin binding capacity decreases with increasing age, suggesting that children can tolerate higher doses of vincristine. Based on these results and previous literature we would suggest that administration of full mg/m^2^ doses to infants from 4 weeks of age may be more appropriate than the currently used mg/kg dosing regimens.

## Supplementary Information

Below is the link to the electronic supplementary material.

Supplementary file1 (DOCX 447 KB)

## Data Availability

The datasets generated during and/or analysed during the current study are available from the corresponding author on reasonable request.

## References

[1] van de Velde ME, Kaspers GL, Abbink FCH, Wilhelm AJ, Ket JCF, van den Berg MH. Vincristine-induced peripheral neuropathy in children with cancer: A systematic review. Crit Rev Oncol Hematol [Internet]. 2017;114:114–30. Available from: 10.1016/j.critrevonc.2017.04.004 10.1016/j.critrevonc.2017.04.004 28477739

[2] Triarico S, Romano A, Attinà G, Capozza MA, Maurizi P, Mastrangelo S, *et al.* Vincristine-Induced Peripheral Neuropathy (VIPN) in Pediatric Tumors: Mechanisms, Risk Factors, Strategies of Prevention and Treatment. Int J Mol Sci [Internet]. 2021;22(8):4112. Available from: https://www.mdpi.com/1422-0067/22/8/4112 10.3390/ijms22084112 PMC8073828 33923421

[3] Legha SS. Vincristine Neurotoxicity. Med Toxicol [Internet]. 1986;1(6):421–7. Available from: http://link.springer.com/10.1007/BF03259853 10.1007/BF03259853 3540519

[4] Teva. Summary of product characteristics: Vincristine sulphate (NL). 2020; Available from: https://www.geneesmiddeleninformatiebank.nl/smpc/h100081_smpc.pdf

[5] Pieters R, Schrappe M, De Lorenzo P, Hann I, De Rossi G, Felice M, *et al.* A treatment protocol for infants younger than 1 year with acute lymphoblastic leukaemia (Interfant-99): an observational study and a multicentre randomised trial. Lancet [Internet]. 2007;370(9583):240–50. Available from: https://linkinghub.elsevier.com/retrieve/pii/S014067360761126X 10.1016/S0140-6736(07)61126-X 17658395

[6] Chi SN, Gardner SL, Levy AS, Knopp EA, Miller DC, Wisoff JH (2004). Feasibility and response to induction chemotherapy intensified with high-dose methotrexate for young children with newly diagnosed high-risk disseminated medulloblastoma. J Clin Oncol.

[7] Rubie H, Coze C, Plantaz D, Munzer C, Defachelles AS, Bergeron C (2003). Localised and unresectable neuroblastoma in infants: Excellent outcome with low-dose primary chemotherapy. Br J Cancer.

[8] Rubie H, De Bernardi B, Gerrard M, Canete A, Ladenstein R, Couturier J, *et al.* Excellent outcome with reduced treatment in infants with nonmetastatic and unresectable neuroblastoma without MYCN amplification: Results of the prospective INES 99.1. J Clin Oncol. 2011;29(4):449–55. 10.1200/JCO.2010.29.5196 21172879

[9] Raney RB, Walterhouse DO, Meza JL, Andrassy RJ, Breneman JC, Crist WM (2011). Results of the Intergroup Rhabdomyosarcoma Study Group D9602 protocol, using vincristine and dactinomycin with or without cyclophosphamide and radiation therapy, for newly diagnosed patients with low-risk embryonal rhabdomyosarcoma: A report from the soft. J Clin Oncol.

[10] Green DM, Cotton CA, Malogolowkin M, Breslow NE, Perlman E, Miser J, *et al.* Treatment of Wilms tumor relapsing after initial treatment with vincristine and actinomycin D: A report from the National Wilms Tumor Study Group. Pediatr Blood Cancer [Internet]. 2007;48(5):493–9. Available from: 10.1002/pbc.20822 10.1002/pbc.20822 16547940

[11] Nijstad AL, Barnett S, Lalmohamed A, Bérénos IM, Parke E, Carruthers V, *et al.* Clinical pharmacology of cytotoxic drugs in neonates and infants: Providing evidence-based dosing guidance. Eur J Cancer [Internet]. 2022;164:137–54. Available from: https://linkinghub.elsevier.com/retrieve/pii/S0959804921012016 10.1016/j.ejca.2021.11.001 PMC8914347 34865945

[12] Barnett S, Hellmann F, Parke E, Makin G, Tweddle DA, Osborne C, *et al.* Vincristine dosing, drug exposure and therapeutic drug monitoring in neonate and infant cancer patients. Eur J Cancer [Internet]. 2021 Oct; Available from: https://linkinghub.elsevier.com/retrieve/pii/S0959804921006092 10.1016/j.ejca.2021.09.014 PMC8914346 34657763

[13] Crom WR, de Graaf SS, Synold T, Uges DR, Bloemhof H, Rivera G, *et al.* Pharmacokinetics of vincristine in children and adolescents with acute lymphocytic leukemia. J Pediatr [Internet]. 1994;125(4):642–9. Available from: http://www.ncbi.nlm.nih.gov/pubmed/7931891 10.1016/s0022-3476(94)70027-3 7931891

[14] Gidding CEM, Meeuwsen-de Boer GJ, Koopmans P, Uges DRA, Kamps WA, de Graaf SS. Vincristine pharmacokinetics after repetitive dosing in children. Cancer Chemother Pharmacol [Internet]. 1999;44(3):203–9. Available from: http://www.ncbi.nlm.nih.gov/pubmed/10453721 10.1007/s002800050968 10453721

[15] Frost BM, Lönnerholm G, Koopmans P, Abrahamsson J, Behrendtz M, Castor A, *et al.* Vincristine in childhood leukaemia: no pharmacokinetic rationale for dose reduction in adolescents. Acta Paediatr [Internet]. 2003 May;92(5):551–7. Available from: http://www.ncbi.nlm.nih.gov/pubmed/12839283 12839283

[16] Guilhaumou R, Simon N, Quaranta S, Verschuur A, Lacarelle B, Andre N (2011). Population pharmacokinetics and pharmacogenetics of vincristine in paediatric patients treated for solid tumour diseases. Cancer Chemother Pharmacol.

[17] Moore AS, Norris R, Price G, Nguyen T, Ni M, George R (2011). Vincristine pharmacodynamics and pharmacogenetics in children with cancer: A limited-sampling, population modelling approach. J Paediatr Child Health.

[18] De Graaf SSN, Bloemhof H, Vendrig DEMM, Uges DRA. Vincristine disposition in children with acute lymphoblastic leukemia. Med Pediatr Oncol [Internet]. 1995;24(4):235–40. Available from: 10.1002/mpo.2950240405 10.1002/mpo.2950240405 7700168

[19] Groninger E, Meeuwsen-de Boer T, Koopmans P, Uges D, Sluiter W, Veerman A, *et al.* Pharmacokinetics of Vincristine Monotherapy in Childhood Acute Lymphoblastic Leukemia. Pediatr Res [Internet]. 2002;52(1):113–8. Available from: http://www.nature.com/doifinder/10.1203/00006450-200207000-00021 10.1203/00006450-200207000-00021 12084857

[20] Plasschaert SLA, Groninger E, Boezen M, Kema I, De Vries EGE, Uges D (2004). Influence of functional polymorphisms of the MDR1 gene on vincristine pharmacokinetics in childhood acute lymphoblastic leukemia. Clin Pharmacol Ther.

[21] Lönnerholm G, Frost B-M, Abrahamsson J, Behrendtz M, Castor A, Forestier E, *et al.* Vincristine pharmacokinetics is related to clinical outcome in children with standard risk acute lymphoblastic leukemia. Br J Haematol [Internet]. 2008;142(4):616–21. Available from: 10.1111/j.1365-2141.2008.07235.x 10.1111/j.1365-2141.2008.07235.x 18537965

[22] Lee CM, Zane NR, Veal G, Thakker DR (2019). Physiologically Based Pharmacokinetic Models for Adults and Children Reveal a Role of Intracellular Tubulin Binding in Vincristine Disposition. CPT Pharmacometrics Syst Pharmacol.

[23] Gout PW, Wijcik LL, Beer CT (1978). Differences between vinblastine and vincristine in distribution in the blood of rats and binding by platelets and malignant cells. Eur J Cancer.

[24] Urien S, Brée F, Breillout F, Bastian G, Krikorian A, Tillement JP. Vinorelbine high-affinity binding to human platelets and lymphocytes: distribution in human blood. Cancer Chemother Pharmacol [Internet]. 1993;32(3):231–4. Available from: 10.1007/BF00685841 10.1007/BF00685841 8500229

[25] Secret CJ, Hadfield JR, Beer CT. Studies on the binding of [3H]vinblastine by rat blood platelets in vitro. Effects of colchicine and vincristine. Biochem Pharmacol. 1972;21(11):1609–24. 10.1016/0006-2952(72)90311-5 4646786

[26] Hebden HF, Hadfield JR, Beer CT (1970). The binding of vinblastine by platelets in the rat. Cancer Res.

[27] Sethi VS, Jackson DV, White DR, Richards F, Stuart JJ, Muss HB, *et al.* Pharmacokinetics of vincristine sulfate in adult cancer patients. Cancer Res [Internet]. 1981;41(9 Part 1):3551 LP – 3555. Available from: http://cancerres.aacrjournals.org/content/41/9_Part_1/3551.abstract 7260915

[28] Owellen RJ, Hartke CA. The pharmacokinetics of 4-acetyl tritium vinblastine in two patients. Cancer Res [Internet]. 1975;35(4):975–80. Available from: http://www.ncbi.nlm.nih.gov/pubmed/1116154 1116154

[29] Cuenca-Zamora EJ, Ferrer-Marín F, Rivera J, Teruel-Montoya R (2019). Tubulin in Platelets: When the Shape Matters. Int J Mol Sci.

[30] van de Velde ME, Panetta JC, Wilhelm AJ, van den Berg MH, van der Sluis IM, van den Bos C (2020). Population pharmacokinetics of vincristine related to infusion duration and peripheral neuropathy in pediatric oncology patients. Cancers (Basel).

[31] Damen CWN, Israëls T, Caron HN, Schellens JHM, Rosing H, Beijnen JH. Validated assay for the simultaneous quantification of total vincristine and actinomycin-D concentrations in human EDTA plasma and of vincristine concentrations in human plasma ultrafiltrate by high-performance liquid chromatography coupled with tandem ma. Rapid Commun Mass Spectrom [Internet]. 2009;23(6):763–74. Available from: 10.1002/rcm.3938 10.1002/rcm.3938 19204931

[32] Israels T, Damen CWN, Cole M, van Geloven N, Boddy A V., Caron HN, *et al.* Malnourished Malawian patients presenting with large Wilms tumours have a decreased vincristine clearance rate. Eur J Cancer [Internet]. 2010;46(10):1841–7. Available from: 10.1016/j.ejca.2010.03.002 10.1016/j.ejca.2010.03.002 20382523

[33] van der Heijden LT, Gebretensae A, Thijssen B, van Andel L, Nijstad AL, Wang Y, *et al.* A highly sensitive bioanalytical method for the quantification of vinblastine, vincristine, vinorelbine and 4-O-deacetylvinorelbine in human plasma using LC-MS/MS. J Pharm Biomed Anal [Internet]. 2022;114772. Available from: https://linkinghub.elsevier.com/retrieve/pii/S0731708522001935 10.1016/j.jpba.2022.114772 35462284

[34] Royal College of Paediatrics and Child Health. UK-WHO Growth charts [Internet]. [cited 2021]. Available from: https://www.rcpch.ac.uk/resources/growth-charts

[35] Du Bois D, Du Bois EF. A formula to estimate the approximate surface area if height and weight be known. 1916. Nutrition. 1989;5(5):303–11; discussion 312. 2520314

[36] West GB, Brown JH, Enquist BJ. A general model for the origin of allometric scaling laws in biology. Science [Internet]. 1997;276(5309):122–6. Available from: http://www.ncbi.nlm.nih.gov/pubmed/9082983 10.1126/science.276.5309.122 9082983

[37] Bergstrand M, Hooker AC, Wallin JE, Karlsson MO. Prediction-corrected visual predictive checks for diagnosing nonlinear mixed-effects models. AAPS J [Internet]. 2011;13(2):143–51. Available from: http://www.ncbi.nlm.nih.gov/pubmed/21302010 10.1208/s12248-011-9255-z PMC3085712 21302010

[38] Dosne AG, Bergstrand M, Karlsson MO (2017). An automated sampling importance resampling procedure for estimating parameter uncertainty. J Pharmacokinet Pharmacodyn.

[39] Lindbom L, Ribbing J, Jonsson EN. Perl-speaks-NONMEM (PsN)--a Perl module for NONMEM related programming. Comput Methods Programs Biomed [Internet]. 2004;75(2):85–94. Available from: http://www.ncbi.nlm.nih.gov/pubmed/15212851 10.1016/j.cmpb.2003.11.003 15212851

[40] Boeckmann AJ, Sheiner LB, Beal SL (2011). NONMEM User Guide. NONMEM Part.

[41] Keizer RJ, van Benten M, Beijnen JH, Schellens JHM, Huitema ADR. Piraña and PCluster: a modeling environment and cluster infrastructure for NONMEM. Comput Methods Programs Biomed [Internet]. 2011;101(1):72–9. Available from: http://www.ncbi.nlm.nih.gov/pubmed/20627442 10.1016/j.cmpb.2010.04.018 20627442

[42] RC Team. R: a language and environment for statistical computing. R Found Stat Comput Vienna. 2009

[43] Oda E, Nakamura Y, Yamamoto M, Kojiro M. Immunohistochemical Distribution of Tubulin beta II in Human Normal and Neoplastic Tissues. Kurume Med J [Internet]. 2005;52(4):117–25. Available from: http://www.jstage.jst.go.jp/article/kurumemedj/52/4/52_4_117/_article 10.2739/kurumemedj.52.117 16639982

[44] Leandro-García LJ, Leskelä S, Landa I, Montero-Conde C, López-Jiménez E, Letón R, *et al.* Tumoral and tissue-specific expression of the major human β-tubulin isotypes. Cytoskeleton [Internet]. 2010;67(4):214–23. Available from: 10.1002/cm.20436 10.1002/cm.20436 20191564

[45] Leandro-García LJ, Leskelä S, Inglada-Pérez L, Landa I, de Cubas AA, Maliszewska A, *et al.* Hematologic β-Tubulin VI Isoform Exhibits Genetic Variability That Influences Paclitaxel Toxicity. Cancer Res [Internet]. 2012;72(18):4744–52. Available from: 10.1158/0008-5472.CAN-11-2861 10.1158/0008-5472.CAN-11-2861 22805305

