# Supplementary material for: A Population Pharmacokinetic Modelling Approach to Unravel the Complex Pharmacokinetics of Vincristine in Children
Source: Pharm Res. 2022 Aug 19;39(10):2487–95. doi: 10.1007/s11095-022-03364-1 (PMC9556337; doi:10.1007/s11095-022-03364-1)
Supplement: Supplementary file 1 — Supplementary file1 (DOCX 447 KB) [file 11095_2022_3364_MOESM1_ESM.docx]

**Supplementary material**

**A population pharmacokinetic modelling approach to unravel the complex pharmacokinetics of vincristine in children**

A. Laura Nijstad, Wan-Yu Chu, Evelien de Vos-Kerkhof, Catherine F. Enters-Weijnen, Mirjam E. van de Velde, Gertjan J.L. Kaspers, Shelby Barnett, Gareth J. Veal, Arief Lalmohamed, C. Michel Zwaan, Alwin D.R. Huitema

**Table S1.** Differential Equations for the mass transport of vincristine between the compartments

| **Compartment** | **Differential equation describing compartment** |
| --- | --- |
| Vincristine central | $\frac{dA(Vc)}{dt}=-\frac{CL}{Vc}\times A\left( Vc \right)-k_{on}\times A\left( Vc \right)\times\left( 1-\frac{A\left( bound \right)}{B_{max}} \right)+k_{off}\times A(bound)-\frac{Q}{Vc}\times A\left( Vc \right)+\frac{Q}{Vp}\times A(Vp)$ |
| Vincristine peripheral | $\frac{dA(Vp)}{dt}=\frac{Q}{Vc}\times A\left( Vc \right)-\frac{Q}{Vp}\times A(Vp)$ |
| Saturable binding to  β-tubulin | $\frac{dA(bound)}{dt}=k_{on}\times A\left( Vc \right)\times\left( 1-\frac{A\left( bound \right)}{B_{max}} \right)-k_{off}\times A(bound)$ |

*A(n)* Amount in compartment n; *B_max_* Maximal binding capacity; *CL* Clearance; *k_off_* Dissociation rate constant; *k_on_* Association rate constant; *Q* Intercompartmental clearance; *Vc* Vincristine central compartment; *Vp* Vincristine peripheral compartment.


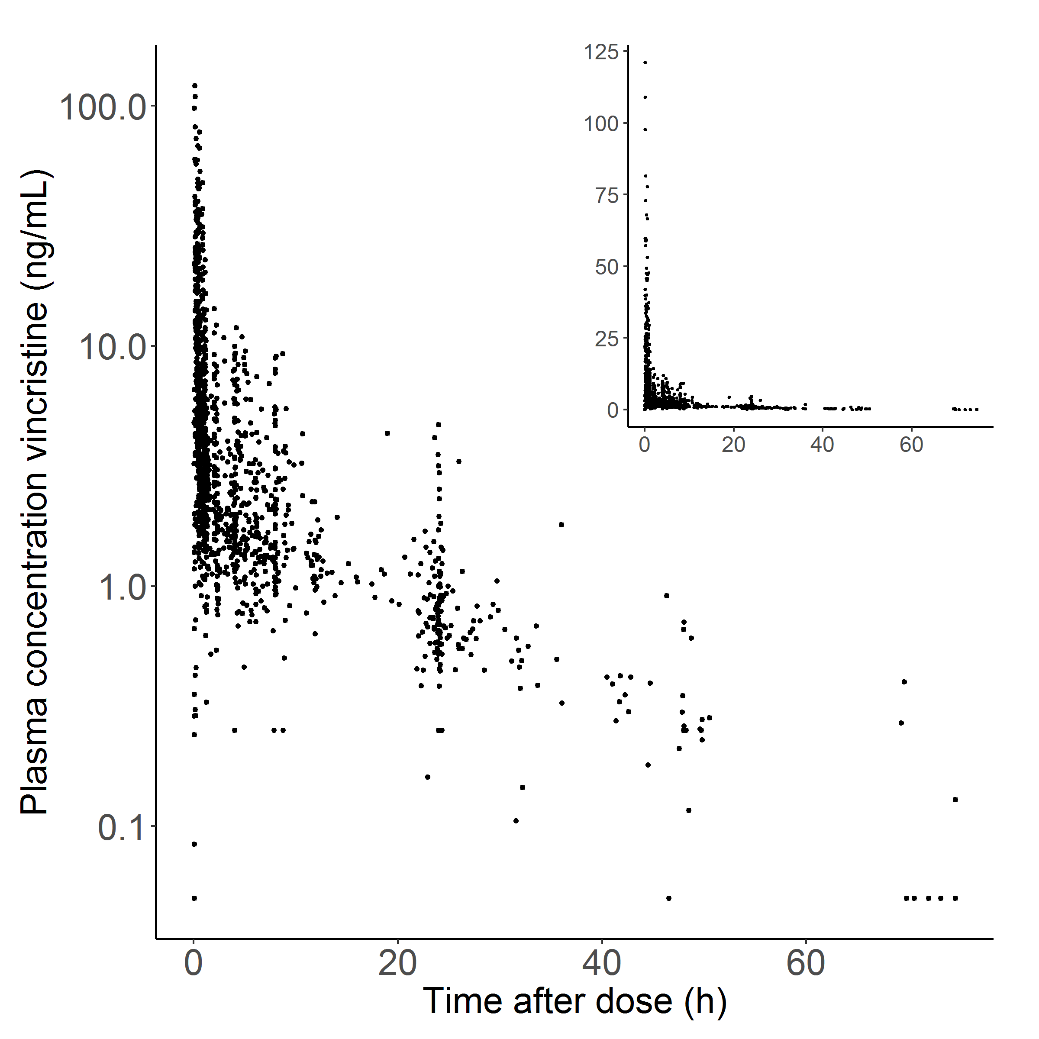


**Figure S1.** Vincristine plasma concentrations versus time after dose on a logarithmic and linear scale.

**
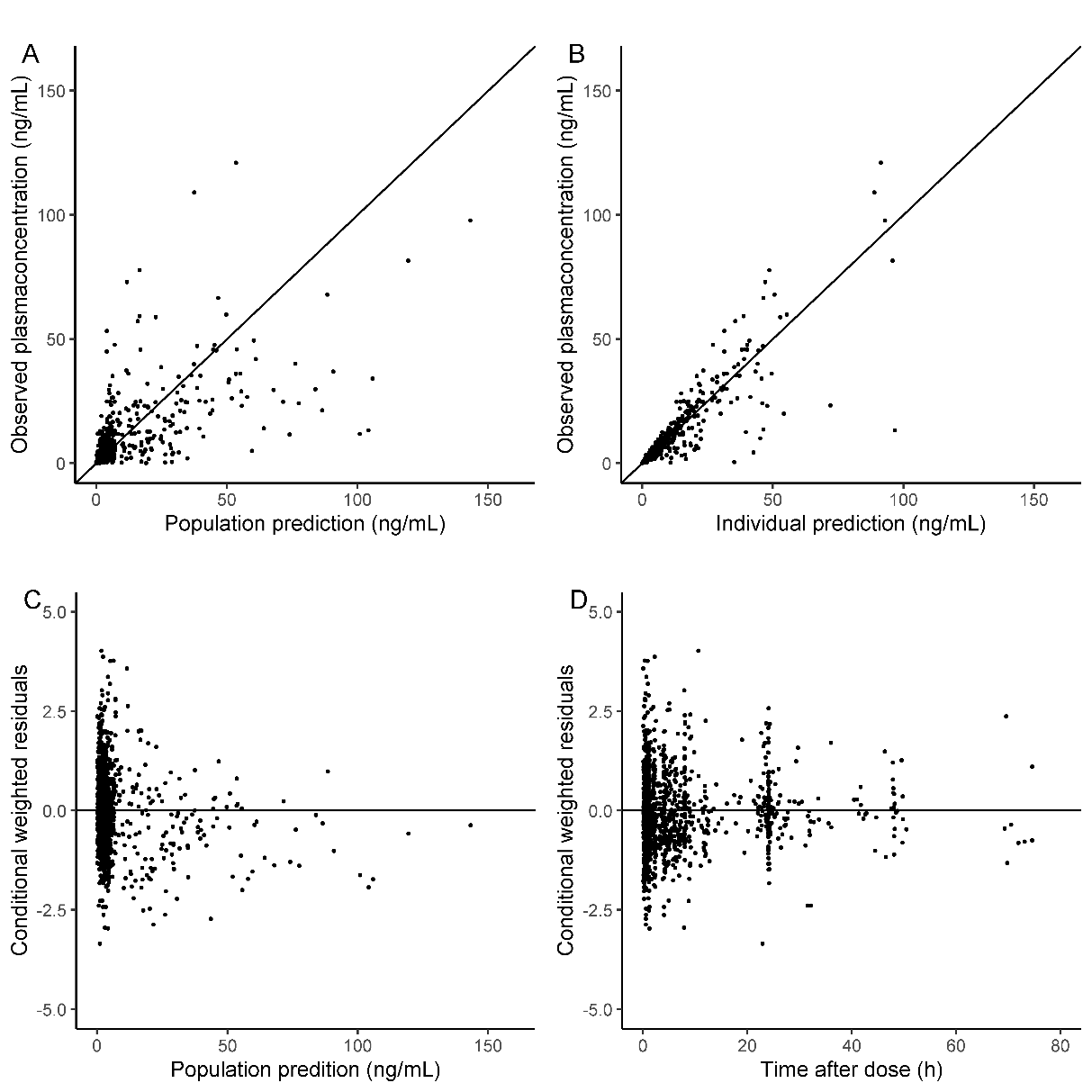
**

**Figure S2.** Goodness-of-fit plots

**
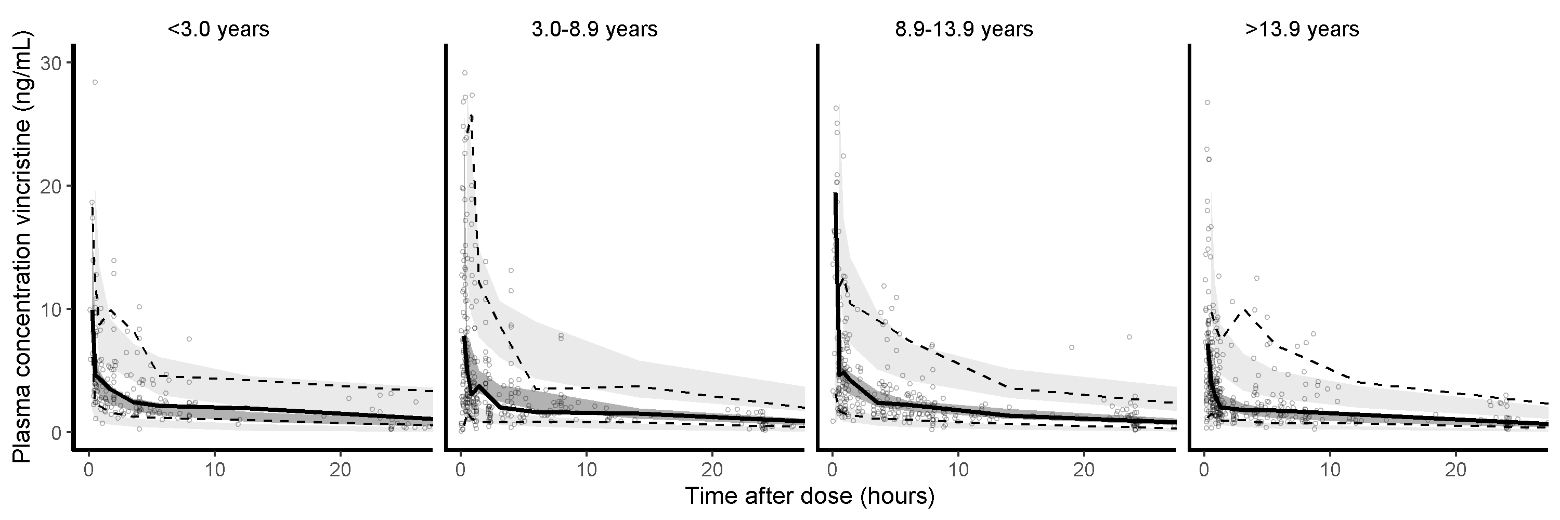
**

**Figure S3.** Age stratified prediction-corrected visual predictive check. Black lines depict the observed median (solid) and 5% and 95% percentile (dashed) concentrations. Dark- and light-grey areas represent 90% prediction intervals of the simulated mean and the 5 and 95% percentiles, respectively. Round dots represent observations.
